# Identifying complications requiring re-operation following primary hip or knee arthroplasty: a consecutive series of 98 patients

**DOI:** 10.1186/s12891-018-2005-y

**Published:** 2018-03-27

**Authors:** Bill Reynolds, Nick Maister, Stephen D. Gill, Shaun Waring, Peter Schoch, Sally Beattie, Andrew Thomson, Richard S. Page

**Affiliations:** 1 Physiotherapy Department, Barwon Health, Geelong, VIC 3220 Australia; 2 Orthopaedic Department, Barwon Health, Geelong, Victoria 3220 Australia; 3 Barwon Centre for Orthopaedic Research and Education (B-CORE), St John of God Hospital and Barwon Health, Geelong, VIC 3220 Australia; 4 School of Medicine, Deakin University, Waurn Ponds, VIC 3220 Australia

**Keywords:** Arthroplasty, Complications, Revision surgery, Hip, Knee

## Abstract

**Background:**

The number of hip and knee arthroplasties completed is expected to double over the next decade. In public hospitals, regular post-arthroplasty orthopaedic review has commonly occurred for the duration of a patient’s life, which requires substantial outpatient resources. However, there is limited evidence regarding the utility of these reviews for identifying complications. The current study investigated when and where complications requiring re-operation were identified following primary hip or knee arthroplasty.

**Methods:**

The medical records of all patients requiring re-operation for complications following primary hip arthroplasty (*n* = 48, 2004 to 2015) or knee primary arthroplasty (*n* = 50, 1998 to 2015) at a large regional health service were evaluated. Data were extracted by one of four investigators using a standardised electronic data extraction tool. Variables of interest included the health setting where the complication was initially identified, how long following the original operation the complication was identified and whether the complication was symptomatic.

**Results:**

Routine post-arthroplasty orthopaedic appointments identified 15 (15.3%) complications requiring re-operation; all were identified in the first-year post-surgery. For each complication identified in the first-year post-surgery, approximately 1000 orthopaedic outpatient appointments were required. After the first year, all complications were identified in Emergency Departments (*n* = 30, 30.6%), General Practice (*n* = 24, 24.5%) or non-routine orthopaedic outpatient appointments (*n* = 19, 19.4%). All patients with complications reported symptoms.

**Conclusions:**

Routine post-arthroplasty review appointments were an inefficient mechanism for identifying complications requiring re-operation more than one year following surgery. Public health services should consider assessing and redesigning post-arthroplasty review services to reduce the burden on patients and the demand for outpatient appointments.

## Background

The number of referrals to public orthopaedic outpatient services are increasing, and waiting times for appointments often exceed recommended targets [1, 2]. Review appointments following hip or knee arthroplasty constitute a large number of appointments. For example, our health service completed approximately 1600 routine hip or knee post-arthroplasty review appointments in 2015 [3]; a figure predicted to increase as the number of hip and knee replacements performed per annum is expected to double in the next decade [4].

The primary purpose of post-arthroplasty review appointments is to identify post-operative complications, such as aseptic prosthetic loosening, that might lead to catastrophic failure if untreated. The frequency of post-arthroplasty review varies between surgeons [5–8]. The Arthroplasty Society of Australia Position Statement indicated that routine reviews following uncomplicated primary arthroplasty in low risk patients and prostheses should take place 2–6 months post implantation, and then at 1–2 years, 5 years and then biennially thereafter [4]. Considering only arthroplasties that occurred in Australian public hospitals in 2016, [9] this review schedule requires approximately 64,000 outpatient appointments in 2017-8 to review the 32,000 primary hip and knee arthroplasties that occurred in 2016; additional appointments would be required to review patients who are 5 years or more following surgery. However, there is little empirical evidence to indicate how often post-arthroplasty review appointments should occur or whether they are necessary at all [2, 5, 10]. The review schedule creates a high burden and cost to patients, families, health services and arthroplasty surgeons. Surgeons cannot regularly review all joint replacement patients without compromising their ability to manage increasing numbers of new patients [4]. Regular review of asymptomatic patients might also contribute to high non-attendance rates amongst this cohort of patients [5, 11].

The current study investigated whether routine orthopaedic outpatient appointments after primary hip or knee arthroplasty were responsible for identifying post-operative complications. Specifically, we aimed to determine 1) the healthcare setting where complications requiring re-operation were initially identified 2) how long after surgery the complications were identified, 3) the type of complication/s identified and 4) the symptomatic and radiological indicators of complications.

## Methods

### Setting

The study was conducted at [removed for anonymous review] (UHG), a 432-bed regional teaching hospital in [removed for anonymous review] that is the only publicly funded hospital in the region. The UHG Orthopaedic Unit currently comprises 8 lower limb arthroplasty surgeons that performed 292 primary hip arthroplasties and 154 primary knee arthroplasties in 2015 [3]. At the time of the study, post-operative *routine* reviews were conducted at the hospital in each surgeon’s weekly outpatient clinic. Patients could be seen post-operatively by the surgeon, orthopaedic registrar or a supervised junior doctor at 6 weeks, 3 months and 12 months, then at 5 and 8 years, and then every two years thereafter. Hip hemiarthroplasties were reviewed in the same manner, provided patients were community ambulant preceding surgery. More frequent *non-routine* reviews occurred at the surgeon’s discretion if there were concerns of increased risk of complications, for example, for prostheses with known higher revision rates. The decision to organise non-routine reviews could be made at any time, including immediately following surgery.

### Study design

**Fig. 1:**
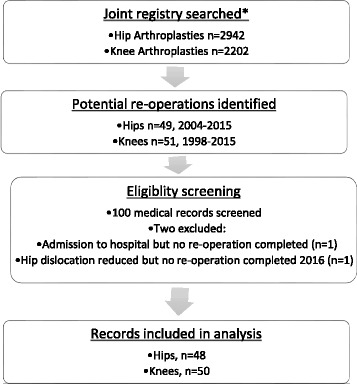
Identification and eligibility screening of records. *database included all arthroplasties including elective, post-trauma, total and partial replacements and re-operations between 1 Jan 1998 to 30 Sept 2015 for knees and between 1 Jan 2004 to 30 Sept 2015 for hips

We planned to retrospectively analyse 100 consecutive primary hip or knee arthroplasties that required re-operation. All hip re-operations that occurred at UHG between 2004 and 2015 (*n* = 49) and all knee re-operations that occurred at UHG between 1998 and 2015 (*n* = 51) were screened for eligibility (see Fig. 1). Including 100 consecutive re-operations was a pragmatic decision based on our ability to identify arthroplasties requiring re-operation (described below) and the resources available to complete the review. Two patients were subsequently excluded from the final data set (see Fig. 1).

### Participants

Eligible participants had undergone primary hip or knee joint arthroplasty at UHG followed by re-operation at UHG. Arthroplasty included total or partial replacement including resurfacing, and a variety of prosthesis types were used at the discretion of each surgeon over the study period. Re-operation included major revision (replacement of components fixed to bone), minor revision (replacement of components not fixed to bone) or washout. Patients with joint dislocation were included only if they subsequently required revision surgery (e.g. patients whose hip dislocations were reduced and had no further orthopaedic intervention were excluded). Patients who underwent multiple revisions had only their first revision included in the analysis.

### Data collection, collation and analysis

Data were extracted from the organisation’s joint replacement registry, which prospectively collects pre-operative and post-operative demographic and outcome data for all people undergoing hip or knee arthroplasty at UHG [12]. Registry data was collected according to the organisation’s routine post-arthroplasty review schedule. Approximately 14% of primary hip arthroplasties and 14% of primary knee arthroplasties have been lost to follow-up since 2004 and 1998 respectively [3]. People requiring re-operation following hip or knee arthroplasty were identified from the joint replacement registry.

The medical records of eligible patients were inspected by one of four members of the research team (BR, NM, SW, PS). Each member analysed 25 cases which were arbitrarily assigned by the primary author (BR). Data extractors were trained by the primary author to collect data in a standardised manner using an electronic data collection template located in the REDCap data management system, hosted at our health service [13]. A senior orthopaedic surgeon (RP or AT) adjudicated to reach consensus if uncertainty existed regarding the classification of information.

Patient demographic data included age, sex and the American Society of Anesthesiologists (ASA) Score [14]. The healthcare setting where the complication was initially identified was classified as either orthopaedic outpatients (routine or non-routine review), inpatients (acute or subacute during the immediate post-operative period), General Practice (GP), Emergency Departments (ED), or other community setting.

Complications were classified as symptomatic, if symptoms were present preceding the re-operation. Radiological indicators were collected from the radiologist’s report and doctor’s notes in the patient’s hospital medical record. How long after the primary operation the complication was identified was categorised as: less than one year, 1-5 years, 6-10 years and more than 10 years.

Data were analysed using descriptive statistics. The chi-square statistic was used to determine if the proportion of people requiring re-operation that were identified at routine orthopaedic outpatient review appointments was different from the proportion of people identified elsewhere. Metal on metal hip resurfacing arthroplasty (*n* = 9) was reported separately due to previously reported high complication rates and unique monitoring requirements [4].

## Results

### Participant characteristics

Of the 48 hip arthroplasty patients, 36 were elective and 12 were post proximal femur fracture of whom 10 received hemi-arthroplasty. The group’s mean age at the time of re-operation was 70.0 years (SD 14.6), 29 (60.4%) were female and the mean ASA score was 2.3 (SD 0.8, 15 patient’s ASA score was irretrievable). Primary hip arthroplasties were completed 6.6 years (average) prior to re-operation (range 15 days to 26.5 years).

Of the 50 knee elective arthroplasty patients, nine received a unilateral knee replacement. The group’s mean age at the time of re-operation was 73.0 years (SD 8.6), 26 (52.0%) were female and the mean ASA score was 2.5 (SD 0.6, 12 patient’s ASA score was irretrievable). Primary knee arthroplasties were completed 4.9 years (average) prior to re-operation (range 7 days to 19.0 years).

### Re-operations completed

Eighty-two re-operations (83.7%) were revisions: 51 (52.0%) were major revisions including both sides of the joint; 28 (28.6%) were major revisions including one side of the joint; and 3 (3.1%) were minor revisions. Fourteen (14.3%) re-operations were washouts, of which 11 subsequently had revision arthroplasty. One patient (1.0%) had an open reduction and internal fixation for peri-prosthetic fracture and the prosthesis was not replaced. One patient (1.0%) had arthroscopic release and joint manipulation for arthrofibrosis.

The approximate cumulative re-operation rate at the hospital (as of 30 September 2015) was 1.0% for primary hip arthroplasties completed between 2004 and 2015 and 1.7% for primary knee arthroplasties completed between 1998-2015.

### Health setting where complication was initially identified

A significantly larger proportion of people requiring re-operation were identified outside of routine orthopaedic outpatient appointments (84.7%) than at routine appointments (15.3%) (*p* < .01) (see Table 1). Of the people identified at routine orthopaedic review appointments, all were identified in the first-year post-surgery (*n* = 15, 15.3%). Complications were most frequently identified in EDs (*n* = 30, 30.6%.), GP (*n* = 24, 24.5%) and non-routine orthopaedic outpatient reviews (*n* = 19, 19.4%).

**Table 1:** Health service where complication was initially identified

	Routine orthopaedic outpatient review		Non-routine orthopaedic outpatient review	ED^a^	GP	Inpatient^b^
	≤ 1 year	> 1 year				
Hip (*n* = 39)	6 (15.4%)	0	5 (12.8%)	14 (35.9%)	8 (20.5%)	6 (15.4%)
Metal on metal (*n* = 9)	0	0	8 (88.9%)	1 (11.1%)	0	0
Knee (*n* = 50)	9 (18.0%)	0	6 (12.0%)	15 (30.0%)	16 (32.0%)	4 (8.0%)
Total	15 (15.3%)	0	19 (19.4%)	30 (30.6%)	24 (24.5%)	10 (10.2%)

No complications were identified in ‘other community settings’

^a^Emergency Department attached to study hospital, except *n* = 2 that were identified by other EDs and referred to the study hospital;

^b^hospital or rehabilitation during the immediate post-operative period (range 2-19 days)

Between 2004 and 2015, approximately 8500 routine 6-week, 12-week and 1-year post hip-arthroplasty review appointments were scheduled [3]. Between 1998 and 2015, approximately 6500 routine 6-week, 12-week and 1-year post knee-arthroplasty review appointments were scheduled [3]. These appointments identified 15 complications, an approximate yield of 1 complication for every 1000 scheduled appointments.

### Duration following primary arthroplasty that complications were identified

Forty-four (44.9%) patients with complications requiring re-operation were identified in the first year following arthroplasty, which increased to 69 (70.4%) by the end of the 5th year (see Table 2).

**Table 2:** Duration following primary arthroplasty when complication was identified (years)

	≤1 year	> 1 ≤ 6 years	6 ≤ 11 years	> 11 years
Hip (*n* = 39)	19 (48.7%)	4 (10.3%)	8 (20.5%)	8 (20.5%)
Hip metal on metal (*n* = 9)	4 (44.4%)	4 (44.4%)	1 (11.1%)	0
Knee (*n* = 50)	21 (42%)	17 (34%)	9 (18%)	3 (6%)
Total	44 (44.9%)	25 (25.5%)	18 (18.4%)	11 (11.2%)

Note: % = (number of re-operations for time period)/(number of re-operations for that arthroplasty type)

### Types of complications

Aseptic loosening was the most common complication which affected 37 (37.8%) patients, followed by infection (19.4%), fracture (15.3%) and dislocation/instability (13.3%) (see Table 3).

**Table 3:** Types of complications

	Aseptic loosening	Infection	Fracture	Dislocation	Progressive joint disease	Defective prosthesis	Mal-alignment	Other
Hip (*n* = 39)	11 (28.2%)	6 (15.4%)	10 (25.6%)	8 (20.5%)	4^a^ (10.3%)	1^c^ (2.6%)	0	0
Hip metal on metal (*n* = 9)	3 (33.3%)	0	1 (11.1%)	0 (0%)	0	4^d^ (44.4%)	0	1^f^(11.1%)
Knee (*n* = 50)	25 (50.0%)	13 (26.0%)	4 (8.0%)	2 (4.0%)	4^b^ (8.0%)	1^e^ (2.0%)	2 (4.0%)	4^g^ (8.0%)
Total	39 (39.8%)	19 (19.4%)	15 (15.3%)	10 (10.2%)	8 (8.2%)	6 (6.1%)	2 (2.0%)	5 (5.1%)

Note: more than one diagnosis possible (five patients had two diagnoses); % is proportion of patients with complication for that arthroplasty type. ^a^Acetabular disease post hemiarthroplasty; ^b^Patello-femoral disease (*n* = 2) or tibio-femoral disease (*n* = 2) post unilateral replacement; ^c^Articular Surface Replacement (ASR) acetabular system [15]; ^d^ASR resurfacing system [15]; ^e^failure of zimmer rotating hinge; ^f^pain without radiological abnormality; ^g^arthrofibrosis (*n* = 1), loose body (*n* = 1), synovitis without infection (*n* = 1), instability (tibiofemoral subluxation) (*n* = 1)

### Symptoms and radiological features of complications

All patients reported joint related pain prior to re-operation, and some patients reported reduced mobility (*n* = 35, 35.7%), joint locking/clicking/stiffness/giving way (*n* = 20, 20.4%) or erythema/swelling/discharge near the joint (*n* = 19, 19.4%). Osteolysis or prosthetic loosening was the most commonly reported x-ray finding (*n* = 36, 36.7%) (see Table 4). No x-ray abnormality was reported for 30 (30.6%) patients.

**Table 4:** X-ray findings

	No visible abnormality	Loosening/ osteolysis	Fracture	Mal-alignment	Polyethylene wear	Effusion	Progressive joint disease
Hip (*n* = 39)	9 (23.1%)	12 (30.8%)	9 (23.1%)	14 (35.9%)	4 (10.3%)	NA	3 (7.7%)
Hip metal on metal (*n* = 9)	5 (55.6%)	3 (33.3%)	1 (11.1%)	2 (2.2%)	NA	NA	0
Knee (*n* = 50)	16 (32.0%)	21 (42.0%)	4 (8.0%)	9 (18.0%)	3 (6.0%)	7 (14%)	4 (8.0%)
Total	30 (30.6%)	36 (36.7%)	14 (14.3%)	25 (25.5%)	7 (7.1%)	7 (7.1%)	7 (7.1%)

Note: x-ray findings according to radiologist’s report and outpatient notes. More than one x-ray finding possible. % is proportion of patients with finding for that arthroplasty type

## Discussion

Post-arthroplasty review is recommended to occur at regular intervals to identify complications [7, 8, 10, 16]. In the current study, routine post-arthroplasty outpatient appointments were an inefficient mechanism for identifying complications that required re-operation, identifying only 15.3% of complications, all in the first-year post-surgery; 1000 routine appointments were required for every complication identified in the first year following arthroplasty. Given the increasing numbers of people requiring joint replacement, routine post-arthroplasty outpatient appointments, particularly beyond 12 months post-surgery, may place unnecessary demand on public health services and alternative models of care should be considered.

Symptoms, rather than radiographic changes were the primary trigger for re-operation; all participants were symptomatic, and 30.6% had normal x-rays. Consistent with our results, Silverwood et al. [17] found that all 183 patients requiring revision total hip arthroplasties were symptomatic, and Hacking et al [18] found 96.4% of 110 total hip arthroplasties were symptomatic [5, 19]. Radiological signs of implant failure can precede symptoms [7], but these patients are commonly kept under observation until symptoms develop, at which point surgery occurs [2]. Because patients self-initiate professional assessment of their symptoms, usually via GP or an ED, following which referral to orthopaedics can be made, regular review of routine post-arthroplasty patients appears duplicative. General practitioners’ and ED staff’s first-point-of-care role for arthroplasty patients requires they have adequate training, knowledge and skills to assess for complications. Efficient communication and referral pathways to orthopaedic care is also required, such as proposed by HealthPathways [20].

Consistent with recommendations, [4] the results of the current study indicate that surgeon discretion might still be necessary when scheduling review appointments; 19.4% of complications were detected at non-routine review appointments. It is unknown however, whether these patients would have been identified in community settings in a timely fashion if non-routine orthopaedic review had not occurred.

Our health service redesigned its post-arthroplasty review service in response to the current investigation. Low risk patients are reviewed in-person at 6 and 12 weeks post primary arthroplasty and then surveyed via mail-out questionnaire at 1, 5 and 8 years post-operatively and then biennially thereafter. The questionnaire used in our remote review system screens for possible complications, expecting that most patients with complications will be symptomatic. Patients with suspected complications are reviewed by the patient’s surgeon. Surgeons can schedule in-person reviews at any time if they are concerned about possible complications, including from higher risk prostheses. Elsewhere, studies indicate that remote post-arthroplasty review compared to in-person review produces lower financial costs to patients, shorter review times, less travel time, less burden on carers, fewer unscheduled outpatient visits and high levels of patient satisfaction; all without missing complications [21–23]. Modelling indicates substantial cost savings and increased quality-adjusted life years from remote models of care [24, 25].

Our remote review system for routine low-risk post-arthroplasty patients is expected to substantially reduce the number of outpatient appointments occurring at our health service. Modelling indicates that in the first year alone, approximately 450 12-month post-operative review appointments will be avoided. An additional 1000 in-person review appointments will be avoided for patients undergoing five-year or longer post-operative review. Reducing the number of post-arthroplasty review appointments will improve the orthopaedic unit’s capacity to see new patients, without requiring additional resources. Adopting this model of care in all Australian public hospitals could reduce the number of scheduled 12-month post-operative review appointments by up to 32,000 in 2017; additional reductions would occur for patients being reviewed at 5 years or beyond.

Consumer-centered care requires that patient preferences are incorporated into all stages of healthcare decision-making including service re-design [26]. Patient preferences regarding post-arthroplasty review are largely unknown and require investigation. Baxter et al. [27] found that 76 of 105 hip or knee arthroplasty patient wanted long-term follow-up, and Sharareh et al [23] found that 59% of patients preferred remote web-based post-arthroplasty review compared to in-clinic review.

Providing remote care requires organisational resources to survey patients; however, we expect a net benefit in favour of reduced organisation costs, consistent with predictions by others [24]. Employing allied staff to provide remote care might be controversial for some surgeons, [19] but preliminary evidence suggests that allied staff, such as physiotherapists can safely and effectively complete post-arthroplasty reviews [28, 29]. Our new service model is in its infancy; its evaluation, including cost-effectiveness and safety is necessary and ongoing.

### Limitations of the current study

The current study identified complications requiring re-operation from an established regional joint replacement registry which relies on surgeons and staff registering re-operations. Although extensive efforts are made by the orthopaedic research unit to identify all re-operations, it is possible that not all were captured in the database used by the current study. Re-operations might have occurred at other health services that were not included in our database. Our retrospective analysis could only collect information recorded in medical histories, which might be incomplete or inaccurate. We only included patients with complications requiring re-operations following primary arthroplasty; the pathways and outcomes for patients with complications not requiring re-operation remain unknown. The results of the current study might not be generalisable to other health services with different populations, prosthesis types, complication rates or follow-up procedures.

## Conclusion

Hip and knee arthroplasties have low rates of post-operative complications and most review appointments are uneventful with no change in clinical management [21] . The current study is the first to our knowledge to report the healthcare setting where complications requiring re-operation are initially detected. Our results indicate that routine post-arthroplasty review appointments are an inefficient mechanism for identifying complications requiring re-operation. Health services should assess their own services and consider alternate models of post-arthroplasty review to meet patient expectations and help manage current and future demand for orthopaedic outpatient services.

## Abbreviations

ASA: American Society of Anesthesiologists
ED: Emergency Department
GP: General Practitce
OA: Osteoarthritis
UHG: University Hospital Geelong
